# The association of histological and radiological indicators of breast cancer risk.

**DOI:** 10.1038/bjc.1988.244

**Published:** 1988-10

**Authors:** S. Urbanski, H. M. Jensen, G. Cooke, D. McFarlane, P. Shannon, V. Kruikov, N. F. Boyd

**Affiliations:** Ludwig Institute for Cancer Research, Toronto, Canada.

## Abstract

**Images:**


Br. J. Cancer (1988), 58, 474–479 © The Macmillan Press Ltd., 1988

### The association of histological and radiological indicators of breast cancer risk

S. Urbanski[1], H.M. Jensen[2], G. Cooke[3], D. McFarlane[4], P. Shannon[1], V. Kruikov[1] & N.F. Boyd[1]

[1]*Ludwig Institute for Cancer Research, Toronto Branch;* [2]*Department of Pathology, School of Medicine, University of California, Davis;* [3]*Department of Radiology, St. Michael's Hospital, Toronto; and* [4]*Toronto Western Hospital, Toronto, Canada.*

**Summary** Previous work has shown that extensive mammographic dysplasia in women aged less than 50 was strongly associated with breast cancer but that the radiological appearance of ductal prominence was not associated with risk. In the present paper we examine the association between these mammographic signs in the breast and histological patterns in the terminal ductal lobular unit (TDLU), the region of the breast where breast cancer is believed to originate. Surgical biopsies from a consecutive series of women aged less than 50 were reviewed and classified according to the histopathology of the epithelium in the TDLU. Mammograms from the same subjects were independently classified according to the extent of the radiological signs of dysplasia and ductal prominence.

Degree of histopathology and the extent of mammographic dysplasia were associated and atypia of the ductal type was found more frequently in patients with extensive dysplasia. However, the strength and statistical significance of the association varied according to the radiologist who classified the mammograms. No association was found between degree of histopathology and ductal prominence. These results add to the evidence that extensive mammographic dysplasia in women aged less than 50 is a risk factor for breast cancer. They do not indicate that the radiological signs of dysplasia are caused by histological changes in the TDLU.

Several reports indicate that classification of the mammographic pattern of the breast provides information about risk of breast cancer (Wolfe, 1976; Krook *et al.*, 1978; Gravelle *et al.*, 1980; Carlile *et al.*, 1985). Most of these reports have used the classification proposed by Wolfe which is based on the radiological signs of ductal prominence and dysplasia. In this system, in the pattern associated with the lowest risk, designated 'N1', the breast parenchyma is comprised mainly of radiolucent fat, and the highest risk appearance, designated 'DY', by the radiological appearance of 'severe dysplasia'. Two patterns associated with intermediate levels of risk are characterized by the appearance of prominent ducts in the breast parenchyma. These patterns are referred to as 'P1' and 'P2' and differ in the proportion of the breast volume that is occupied by prominent ducts.

In previous work, we have found that mammographic dysplasia was strongly associated with breast cancer, particularly when it extensively involved the breast in women aged less than 50 (Boyd *et al.*, 1982). We found, however, that ductal prominence was only weakly associated with breast cancer risk. These findings are summarized in Table I. The data shown were obtained from a case control study in which 183 cases with unilateral breast cancer were individually age matched with controls attending a screening centre. Mammograms from the non-cancerous breast of the cases were randomly assembled with those from the controls and classified, without knowledge of which came from cases or controls, according to the proportion of the breast volume occupied by the radiological signs of dysplasia and ductal prominence. The data shown in Table I are from women aged less than 50 in whom mammographic dysplasia was found to be most strongly associated with breast cancer. A statistically significant linear trend was found between breast cancer and increasing replacement of the breast volume by the radiological changes of dysplasia, but no association was found between breast cancer and ductal prominence.

While there is an extensive literature describing the non-cancerous histological changes in the breast that are found in association with breast cancer (Cheattle & Cutler, 1931; Foote & Stewart, 1950; Wellings *et al.*, 1975) and the relationship of these changes to risk of breast cancer (Warren, 1940; Kiaer, 1954; Black *et al.*, 1972; Page *et al.*, 1978; Hutchinson *et al.*, 1980; Dupont & Page, 1984), there is little information available about the types of breast histology that occur in association with the radiological changes that are considered to be indicators of breast cancer risk.

The objective of the work reported here was to examine the association between the radiological changes that are associated with an increased risk of breast cancer. Because our previous work has shown that mammographic dysplasia is a risk factor for breast cancer in younger women, we have confined this study to patients aged 50 or less. The histological classification used is based upon the appearance of the epithelium in the terminal ductal lobular unit (TDLU), the region of the breast that is thought to be the site of origin of breast cancer (Wellings *et al.*, 1975).

### Materials and methods

### *Selection of material*

The material analyzed in this study was selected from the files of the Department of Radiology of the Toronto Western Hospital. These files allow the identification of patients who have had both mammograms and biopsy of the breast. We selected a consecutive series of patients in whom both mammograms and a biopsy of the breast had been done and retained them if they were aged 50 or less, the interval between mammogram and biopsy had been less than 12 months, and no diagnosis of invasive cancer of the breast had been made. All patients who met these criteria were retained regardless of the findings on mammography or biopsy.

Histological slides and mammograms were classified separately and independently. Each modality was read 'blindly' with respect to the other, that is radiologists were not provided with any information about biopsy findings and the histology was studied without any knowledge of radiological findings.

Correspondence: N.F. Boyd, Department of Medicine, Princess Margaret Hospital, 500 Sherbourne St., Toronto, Ontario, M4X 1K9, Canada.
Received 2 October 1987; and in revised form, 12 July 1988.

**Table I** Summary of previous work: Association of mammographic dysplasia and ductal prominence with breast cancer[a] in patients aged less than 50

| | *Extent of dysplasia* | | | | | |
|---|---|---|---|---|---|---|
| | *<10%* | *<25%* | *<50%* | *<75%* | *≧75%* | *Total* |
| Controls | 57 | 8 | 4 | 4 | 6 | 80 |
| Cases | 32 | 8 | 11 | 12 | 17 | 80 |
| Total | 89 | 16 | 16 | 16 | 23 | 160 |
| Odds ratio | 1.00 | 1.78 | 3.92 | 5.34 | 5.05 | |
| $\chi^2$ for trend = 18.52; $P$ = 0.00001 | | | | | | |
| | *Extent of ductal prominence* | | | | | |
| Controls | 32 | 30 | 11 | 5 | 2 | 80 |
| Cases | 40 | 20 | 10 | 5 | 5 | 80 |
| Total | 72 | 50 | 21 | 10 | 7 | 160 |
| Odds ratio | 1.00 | 0.53 | 0.73 | 0.80 | 2.0 | |
| $\chi^2$ for trend = 1.40; $P$ = 0.236 | | | | | | |

[a]Adapted from Boyd *et al.* (1982).

*Classification of histology*

Histological classification was made on one slide from each block, stained with haematoxylin and eosin. Each slide was classified according to whether terminal duct lobular unit (TDLU) were present, and if present according to the histopathology of the epithelial cell population in the TDLU. The lesions were all of the so called ductal type commonly called papillomatosis in the United States and epitheliosis in the United Kingdom. The epithelium in the TDLU was classified according to whether it was normal, hyperplastic or atypical. If atypia was present, it was further classified as mild, moderate or severe. Ductal carcinoma *in situ* was classified separately. When more than one histological pattern was present within one biopsy specimen, the patient was classified according to the highest degree of disease seen. The classification used is a minor modification of that described elsewhere (Wellings *et al.*, 1975). Examples of each of the grades of atypia and ductal carcinoma *in situ* are illustrated in Figure 1. Ductal hyperplasia is not illustrated. It displays moderately distended ductules lined by 2–3 cell layered epithelium with prominent cytoplasmic luminal 'snouts'. No examples of lobular hyperplasia or atypia were encountered.

*Classification of radiology*

Films were separated from previous reports of either pathology or radiology findings, placed in new envelopes, and independently read by two radiologists who classified them according to proportion of the breast volume occupied by signs of dysplasia or ductal prominence. Examples of the classification as applied to dysplasia are shown in Figure 2.

*Statistical procedures*

Concordance between radiological and histological methods of classification was assessed with Kendall's tau statistic (Kendall, 1955). This statistic provides a measure of concordance between ranked ordinal data and can assume a value between $-1$ and $+1$, indicating respectively perfect inverse and positive concordance. Proportions were compared with the chi-squared test and a linear trend in proportions using the chi-squared test for trend described by Bartholomew (1959). Odds ratios were calculated using standard methods (Fleiss, 1973).

### Results

*Characteristics of subjects*

The mean age of the subjects included in the study was 42.5 years, range 30 to 50 years, standard deviation 4.96 years.

*Distribution of mammographic and histological findings*

Table II shows the distribution of patients according to the extensiveness of the radiological signs of dysplasia and ductal prominence, and according to the histopathology of the TDLU. According to radiologist 1, 55% of subjects had mammographic dysplasia occupying more than 50% of the breast volume, while in 76% of subjects ductal prominence occupied less than 10% of the breast. Radiologist 2 classified 34% of films as showing dysplasia in less than 50% of the breast and 43% as showing ductal prominence in less than 10% of the breast. About one third of the biopsies showed some evidence of ductal atypia, but only 3 showed severe atypia and 1 showed ductal carcinoma *in situ*. In the analyses that follow, we have initially combined all grades of ductal atypia and ductal carcinoma *in situ* into a single category.

*Association of epithelial grade and mammographic dysplasia*

Table III shows the association between histopathology and the extent of mammographic dysplasia in the breast from which the biopsy was obtained. The histological classifications shown are those of HJ. The radiological classifications shown are those of GC, identified as radiologist 1 in Table III, who also generated the data given in Table I. Only two (10%) of the 20 biopsies from women with less than 10% of breast occupied by signs of dysplasia showed evidence of atypia compared to 17 (45%) of the 40 biopsies from women with radiological dysplasia in more than 75% of the breast volume. Statistical assessment of Table III as a whole showed that increasing degree of histological abnormality and the extent of radiological dysplasia were significantly related (Kendall's tau B = 0.177, standard error = 0.667; t = 2.64; $P < 0.01$).

A more detailed examination of the relationship between histology and radiology is shown at the foot of Table III. The odds ratios shown indicate, in each category of dysplasia, the probability of observing atypia or hyperplasia in a biopsy. The odds ratios were calculated by dividing the ratio of atypical (or hyperplastic) to normal biopsies within each radiological category by the ratio found in the category of least extensive dysplasia.

The data show that there is a statistically significant association between the probability of finding atypia in a biopsy and the extensiveness of dysplasia in the breast from which the biopsy was taken. A biopsy from a breast with more than 75% of its volume occupied by dysplasia was 9 times more likely to show atypia and 3 times more likely to show hyperplasia than a biopsy from a breast with less than 10% of the volume replaced by dysplasia. Despite the statistically significant trend statistic the gradient in risk was not entirely monotonic across categories of mammographic

**Figure 1** **(a)** TDLU shows moderate distension of ductules and is filled with cells. Arrows point to area magnified in (b) (×30). **(b)** The cell population is pleomorphic. This is graded as mild ductal atypia (×190). **(c)** TDLU shows marked distension of its terminal duct and few coarse ductules are noted. Arrows point to area magnified in (d) (×30). **(d)** The cell population is pleomorphic and spaces are irregular. This is graded as moderate ductal atypia (×190). **(e)** Several TDLU show marked distension. Arrows point to area magnified in (f) (×30). **(f)** The cell population shows little variation in size and shape. The spaces are irregular. This is graded as severe ductal atypia (×190). **(g)** Several TDLU display marked distension and a trabecular 'lacy' pattern. Arrows point to area magnified in (h) (×30). **(h)** The cell population is monotonous and the spaces are less irregular. This is graded as ductal carcinoma *in situ* (×190). Magnification Bars: Low Power 1 mm = 30 mm; High Power 0.1 mm = 10 mm.

**Figure 2** These five mammographic patterns correspond to the replacement of the breast by an increasing proportion of radiographic dysplasia. (All views are mediolateral).

dysplasia and a statistically significant increase in risk of atypia was seen in women with 10<25% of the breast occupied by dysplasia ($\chi^2=8.22$; $P=0.004$). In view of the small numbers in this category this finding may be due to chance, statistics notwithstanding.

*Association of histopathology and ductal prominence*

Table IV shows the association between amount of histopathology and the extent of ductal prominence on mammography. Forty-two (37%) of the 112 biopsies from women with less than 10% of breast occupied by signs of ductal prominence showed evidence of atypia compared to 2 (40%) of the 5 biopsies from women with radiological ductal prominence in more than 75% of the breast volume. Statistical assessment of Table IV as a whole showed that degree of histopathology and the extent of radiological ductal prominence were not significantly related (Kendall's tau B$=-0.026$; $t=0.33$; $P>0.50$).

The odds ratios shown at the foot of Table IV show that the probability of observing atypia or hyperplasia in a biopsy was not related to the extent of ductal prominence in the mammogram.

**Table II** Distribution of mammographic and histological findings

| *Extent* | *Dysplasia* | *Ductal prom.* | *Dysplasia* | *Ductal prom.* | *Histology* |
|---|---|---|---|---|---|
| <10% | 20 (13%) | 112 (76%) | 10 (7%) | 63 (43%) | Normal 67 (46%) |
| 10<25% | 14 (9%) | 22 (15%) | 17 (12%) | 22 (15%) | Hyperplasia 26 (18%) |
| 25<50% | 32 (22%) | 6 (4%) | 22 (15%) | 20 (13%) | Atypia (mild) 25 (17%) |
| 50>75% | 41 (28%) | 2 (1.4%) | 23 (16%) | 15 (10%) | Atypia (moderate) 25 (17%) |
| ≧75% | 40 (27%) | 5 (3.4%) | 75 (51%) | 27 (18%) | Atypia (severe) 3 (2%) |
| | | | | | Carcinoma – *in situ* 1 (0.7%) |
| Total | 147 | 147 | 147 | 147 | 147 |

**Table III** Association of histopathology and extent of mammographic dysplasia (radiologist 1)

| *Histology* | *<10%* | *10<25%* | *25<50%* | *50<75%* | *≧75%* | *Total* |
|---|---|---|---|---|---|---|
| Normal | 15 | 6 | 16 | 16 | 14 | 67 |
| Hyperplasia | 3 | 1 | 7 | 6 | 9 | 26 |
| Atypia/Dcis[a] | 2 | 9 | 9 | 19 | 17 | 54 |
| Total | 20 | 14 | 32 | 41 | 40 | 147 |
| *Odds ratios* | | | | | | |
| Normal *vs.* hyperplasia | 1.0 | 0.83 | 2.19 | 1.87 | 3.21 | |
| $\chi^2$ for trend$=1.47$; $P=0.225$ | | | | | | |
| Normal *vs.* atypia | 1.0 | 8.75 | 4.22 | 8.90 | 9.11 | |
| $\chi^2$ for trend$=8.30$; $P=0.004$ | | | | | | |

[a]Dcis = Ductal carcinoma *in situ*.

*Association of atypia and mammographic dysplasia*

Examination of the association between degree atypia on biopsy and the extent of dysplasia on mammography showed that both mild and moderate atypia were found with increasing frequency, the more extensive the radiological signs of dysplasia on mammography were (data not shown). The single example of ductal carcinoma *in situ* occurred in a patient with more than 75% of the breast occupied by radiological changes of dysplasia as did 2 of the 3 cases of severe atypia. The third example of severe atypia was found in a patient with dysplasia in between 50 and 75% of the breast volume.

*Association of atypia and ductal prominence*

No association was found between degree of atypia on biopsy and the extent of ductal prominence on mammography (data not shown). Neither mild or moderate/severe atypia showed any tendency to occur more frequently in biopsies from patients with extensive replacement by ductal prominence.

*Reproducibility of results*

All results were reanalyzed using the classifications of a second radiologist and are shown in tables V and VI. While the results of the second radiologist differed quantitatively from those of radiologist 1, and failed to achieve statistical significance for the association between mammographic dysplasia and atypia, they resemble those of radiologist 1 in showing that ductal hyperplasia with atypia was associated more strongly with the extent of dysplasia than with the extent of ductal prominence. The value for Kendall's tau for the overall association of degree of histopathology with dysplasia was 1.45 ($0.10<P<0.20$), and for ductal prominence was 0.45 ($P<0.50$). The $\chi^2$ for trend for the association of histologic atypia and the extent of dysplasia was 1.9 ($0.10<P<0.25$).

### Discussion

The associations found by radiologist 1 in this study between mammographic and histological indicators of breast cancer risk in women aged 50 or less conform precisely to those expected from the earlier case control study in which this radiologist classified films (Boyd *et al.*, 1982). Mammographic dysplasia was found to be associated with risk of breast cancer and was here found to be associated also with histological changes that confer increased risk. The mammographic signs of ductal prominence were found to have no association with breast cancer risk and were here found to have no association with histological indicators of risk.

Although similar associations were seen using the mammographic classifications of a second radiologist the strength

**Table IV** Association of histopathology and extent of ductal prominence (radiologist 1)

| *Histology* | *<10%* | *10<25%* | *25<50%* | *50<75%* | *≥75%* | *Total* |
|---|---|---|---|---|---|---|
| Normal | 50 | 10 | 3 | 1 | 3 | 67 |
| Hyperplasia | 20 | 6 | 0 | 0 | 0 | 26 |
| Atypia/Dcis[a] | 42 | 6 | 3 | 1 | 2 | 53 |
| Total | 112 | 22 | 6 | 2 | 5 | 147 |
| *Odds ratios* | | | | | | |
| Normal *vs.* hyperplasia | 1.0 | 0.71 | 0.41 | 1.25 | 0.42 | |
| $\chi^2$ for trend=2.94; $P$=0.086 | | | | | | |
| Normal *vs.* atypia | 1.0 | 0.71 | 1.19 | 1.19 | 0.79 | |
| $\chi^2$ for trend=0.01; $P$=0.913 | | | | | | |

[a]Dcis=Ductal carcinoma *in situ*.

**Table V** Association of histopathology and extent of mammographic dysplasia (radiologist 2)

| *Histology* | *<10%* | *10<25%* | *25<50%* | *50<75%* | *≥75%* | *Total* |
|---|---|---|---|---|---|---|
| Normal | 5 | 9 | 10 | 13 | 30 | 67 |
| Hyperplasia | 3 | 2 | 6 | 1 | 14 | 26 |
| Atypia/Dcis[a] | 2 | 6 | 6 | 9 | 31 | 54 |
| Total | 10 | 17 | 22 | 23 | 75 | 147 |
| *Odds ratios* | | | | | | |
| Normal *vs.* hyperplasia | 1.00 | 0.37 | 1.50 | 0.13 | 0.78 | |
| $\chi^2$ for trend=0.00; $P$=0.996 | | | | | | |
| Normal *vs.* atypia | 1.00 | 1.50 | 1.50 | 1.73 | 2.58 | |
| $\chi^2$ for trend=1.9; $P$=0.168 | | | | | | |

[a]Dcis=Ductal carcinoma *in situ*.

**Table VI** Association of histopathology and extent of ductal prominence (radiologist 2)

| *Histology* | *<10%* | *10<25%* | *25<50%* | *50<75%* | *≥75%* | *Total* |
|---|---|---|---|---|---|---|
| Normal | 30 | 7 | 10 | 10 | 10 | 67 |
| Hyperplasia | 10 | 8 | 2 | 2 | 4 | 26 |
| Atypia/Dcis[a] | 23 | 7 | 8 | 3 | 13 | 54 |
| Total | 63 | 22 | 20 | 15 | 27 | 147 |
| *Odds ratios* | | | | | | |
| Normal *vs.* hyperplasia | 1.00 | 3.42 | 0.60 | 0.60 | 1.20 | |
| $\chi^2$ for trend=0.16; $P$=0.69 | | | | | | |
| Normal *vs.* atypia | 1.00 | 1.30 | 1.04 | 0.39 | 1.70 | |
| $\chi^2$ for trend=0.14; $P$=0.71 | | | | | | |

[a]Dcis=Ductal carcinoma *in situ*.

of the association differed and did not reach statistical significance for the second reader.

These results thus provide further evidence that radiological dysplasia is a risk factor for breast cancer, at least in younger women, and provide a biological explanation for the association of this mammographic sign with cancer risk. Further, if the view is correct that breast cancer usually arises in the region of the TDLU, then it would not be expected that a radiological sign that is caused by the deposition of fibrous tissue around major ducts would be associated with risk of breast cancer.

The results of this study resemble those of some previous studies. Ingleby & Gershon-Cohen (1960) in their work on the relationship between histological and radiological appearances of the breast showed that the histological appearance they called 'adenosis', which was characterized by intraductal hyperplasia, was associated with diffuse or nodular opacities on mammography which could be either localized or generalized and which have come to be called 'dysplasia'. The histological entity of 'adenosis' was believed by Ingleby & Gershon-Cohen to resemble the lesions described by earlier workers, including Warren (1940) and Kaier (1954), as being associated with an increased risk of breast cancer.

Wellings & Wolfe (1978), using a similar method of grading the breast epithelium that was used in this study, also reported an association between the higher risk histological patterns and the P2 and DY mammographic patterns that Wolfe had earlier described as risk factors for breast cancer. The finding by these workers that the P2 pattern was related to degree of histopathology thus differs from our findings concerned ductal prominence and may be explained by Wolfe's inclusion in the P2 category of some classes of homogeneous density that other radiologists usually classified as dysplasia (de Waard & Rombach, 1987).

Fisher & coworkers (1978) compared the histology of the breast in women with breast cancer and women with breast cancer and women with fibrocystic disease and were unable to find any epithelial appearance that was particularly associated with any mammographic appearance. However, the selection as a comparison group of women with non-malignant abnormalities sufficiently suspicious to warrant biopsy may have obscured distinctions that would have been present in a less highly selected population.

Bright *et al.* (1988) described an association between mammographic densities and intralobular fibrosis in premenopausal women and epithelial hyperplasia or atypia in postmenopausal women.

None of these studies, however, provide any information about the histological changes that are responsible for the radiological appearance of dysplasia. There is some evidence that they are caused by changes in the stroma of the breast (Fisher *et al.*, 1978), where they appear to be under hormonal control, as evidenced by the changes in the radiological appearance of dysplasia that have been observed after use of the drug Danazol (Asch & Greenblatt, 1977). If the radiological signs of dysplasia are indeed caused by changes in the stroma of the breast, then their association with epithelial atypia and breast cancer risk may represent reactions in different tissues to the environmental agent(s) responsible for breast cancer.

The present study provides no information about the relationship between histological and radiological appearance in women over the age of 50 and contains only very limited data on the distribution of severe epithelial atypia in the TDLU in relation to mammographic pattern. This latter shortcoming is particularly important because the evidence that degree of histopathology is related to cancer risk is especially strong for severe atypia (Dupont & Page, 1985). The frequency of severe atypia in the group of biopsies examined in this study suggests that a sample at least 5 times the size of this one (i.e. a minimum of 700 biopsies) will be required adequately to examine the relation of severe atypia to radiological dysplasia.

Further information about the tissue changes that are responsible for mammographic dysplasia and clarification of the relationship of severe epithelial atypia to mammographic dysplasia may provide improved methods for identifying individuals at increased risk for breast cancer and ultimately provide a basis for the development of preventive strategies.

### References

ASCH, R.M. & GREENBLAT, R.B. (1977). The use of an impeded androgen – Danazol – in the management of benign breast disease. *Am. J. Obstet. Gynecol.*, **127**, 130.

BARTHOLOMEW, D.J. (1959). A test of homogeneity for ordered alternative. *Biometrika*, **46**, 328.

BLACK, M.M., BARCLAY, T.H., CUTLER, S.J., HANKEY, B.F. & ASIRE, A.J. (1972). Association of atypical characteristics of benign breast lesions with subsequent risk of breast cancer. *Cancer*, **29**, 338.

BOYD, N.F., O'SULLIVAN, B., CAMPBELL, J. & 4 others (1982). Mammographic signs as risk factors for breast cancer. *Br. J. Cancer*, **45**, 188.

BRIGHT, R.A., MORRISON, A.S., BRISSON, J. & 4 others (1988). Relationship between mammographic and histologic features of breast tissue in women with benign breast biopsies. *Cancer*, **61:** 266.

CARLILE, T., KOPECKY, K.J., THOMPSON, D.J. & 6 others (1985). Breast cancer prediction and the Wolfe classification of mammographic parenchymal pattern. *J. Amer. Med. Assoc.*, **254**, 1050.

CHEATTLE, G.L. & CUTLER, M. (1931). *Tumors of the Breast: Their Pathology, Symptoms, Diagnosis and Treatment*. Arnold: London.

DUPONT, W.D. & PAGE, D.L. (1984). Risk factors for breast cancer in women with proliferative breast disease. *New Engl. J. Med.*, **312**, 146.

FISHER, E.R., PALEKER, A., KIM, W.S. & REDMOND, C. (1978). The histopathology of mammographic patterns. *Am. J. Clin. Path.*, **69**, 421.

FLEISS, J. (1973). *Statistical Tests for Rates and Proportions*. John Wiley and Sons Ltd.: London.

FOOTE, F.W. & STEWART, F.W. (1950). Comparative studies of cancerous versus noncancerous breasts. *Ann. Surg.*, **121**, 6.

GRAVELLE, I.H., BULSTRODE, J.C., WAND, D.Y. & HAYWARD, J.L. (1980). The relation between radiographic features and determinants of risk of breast cancer. *Br. J. Radiology*, **53**, 107.

HUTCHINSON, W.B., THOMAS, D.B., HAMLIN, W.B., ROTH, G.J., PETERSON, A.V. & WILLIAMS, B. (1980). Risk of breast cancer in women with benign disease. *J. Natl Cancer Inst.*, **65**, 13.

INGLEBY, H. & GERSHON-COHEN, J. (1960). *Comparative Anatomy, Pathology and Roentgenology of the Breast*. University of Pennsylvania Press: Philadelphia.

KENDALL, M.G. (1955). *Correlation Methods*. John Wiley and Sons Ltd.: London.

KIAER, W. (1954). *Relation of fibroadenomatosis to cancer of the breast*. Ejnar Munksgaard: Copenhagen.

KROOK, P.M., CARLILE, T., BUSH, W. & HALL, M.H. (1978). Mammographic parenchymal patterns as a risk indicator for prevalent and incident breast cancer. *Cancer*, **41**, 1093.

PAGE, D.L., VANDER ZWAAG, R., ROGERS, L.W., WILLIAMS, L.,T., WALKER, W.E. & HARTMANN, W.H. (1978). Relation between component parts of fibrocystic disease complex and breast cancer. *J. Natl Cancer Inst.*, **61**, 1055.

WARREN, S. (1940). The relation of 'chronic mastitis' to carcinoma of the breast. *Surg. Gynecol. Obstet.*, **71**, 257.

WELLINGS, S.R., JENSEN, H.M. & MARCUM, R.G. (1975). An atlas of the subgross pathology of the human breast with special reference to possible precancerous lesions. *J. Natl Cancer Inst.*, **55**, 231.

WELLINGS, S.R. & WOLFE, J.N. (1978). Correlative studies of the histologic and radiographic appearance of the breast parenchyma. *Radiology*, **129**, 299.

WOLFE, J.N. (1976). Risk for breast cancer development determined by mammographic parenchymal pattern. *Cancer*, **37**, 2486.

DE WAARD, F. & ROMBACH, J.J. (1987). The relationship between Wolfe's classification of mammograms, accepted breast cancer risk factors, and the incidence of breast cancer. *Am. J. Epidemiol.*, **125**, 171 (Letter).


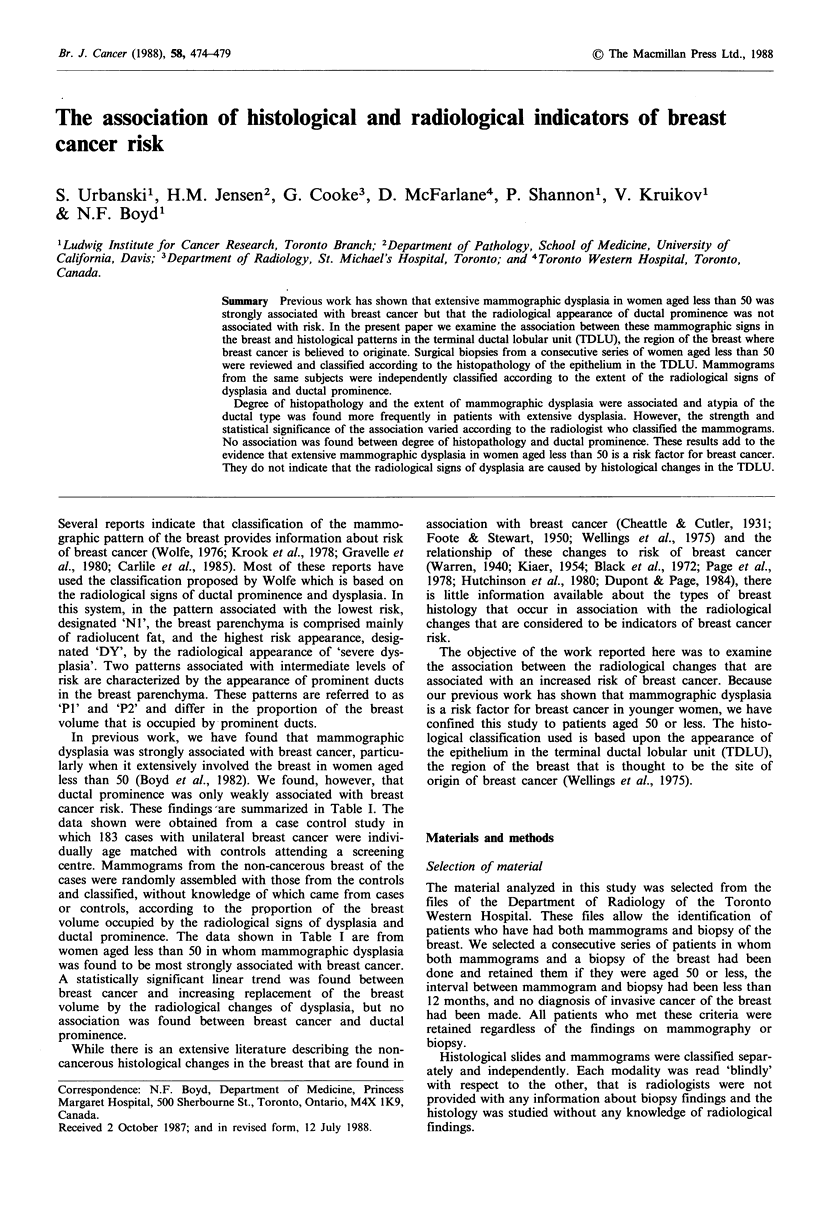

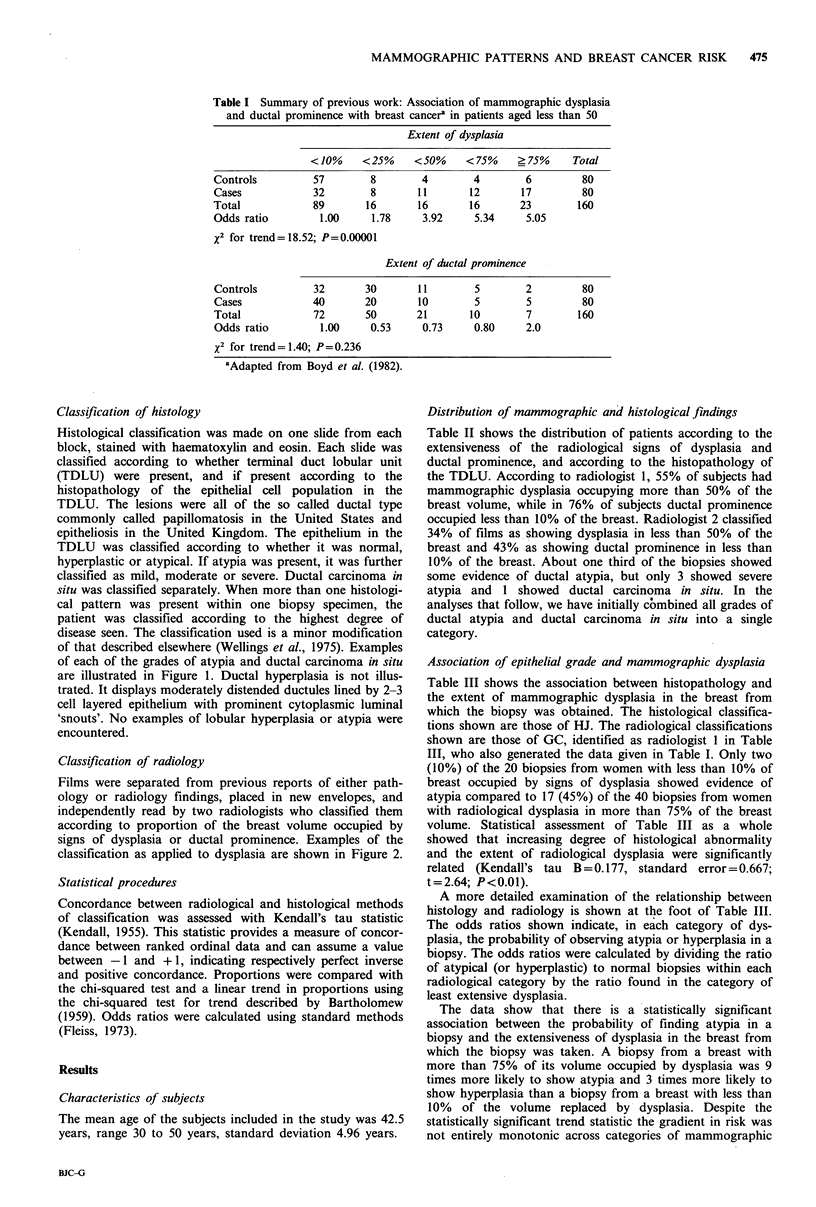

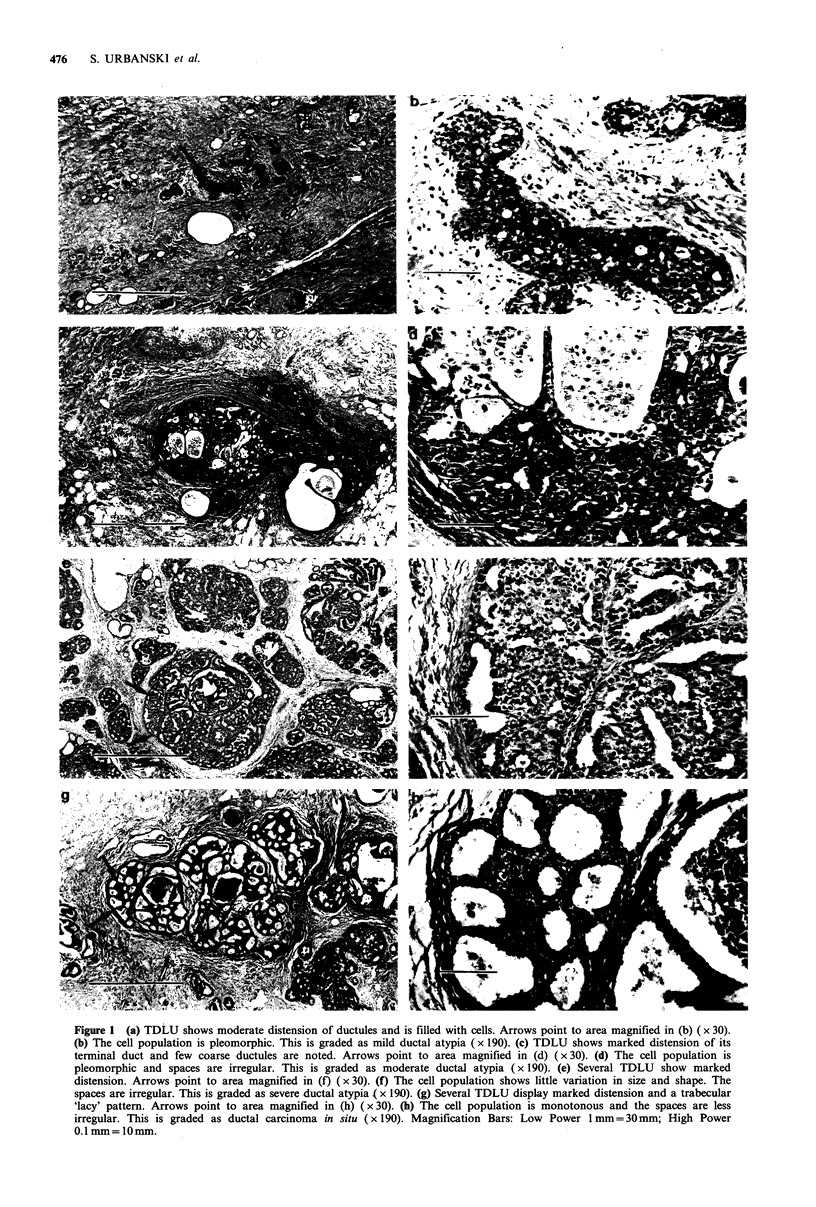

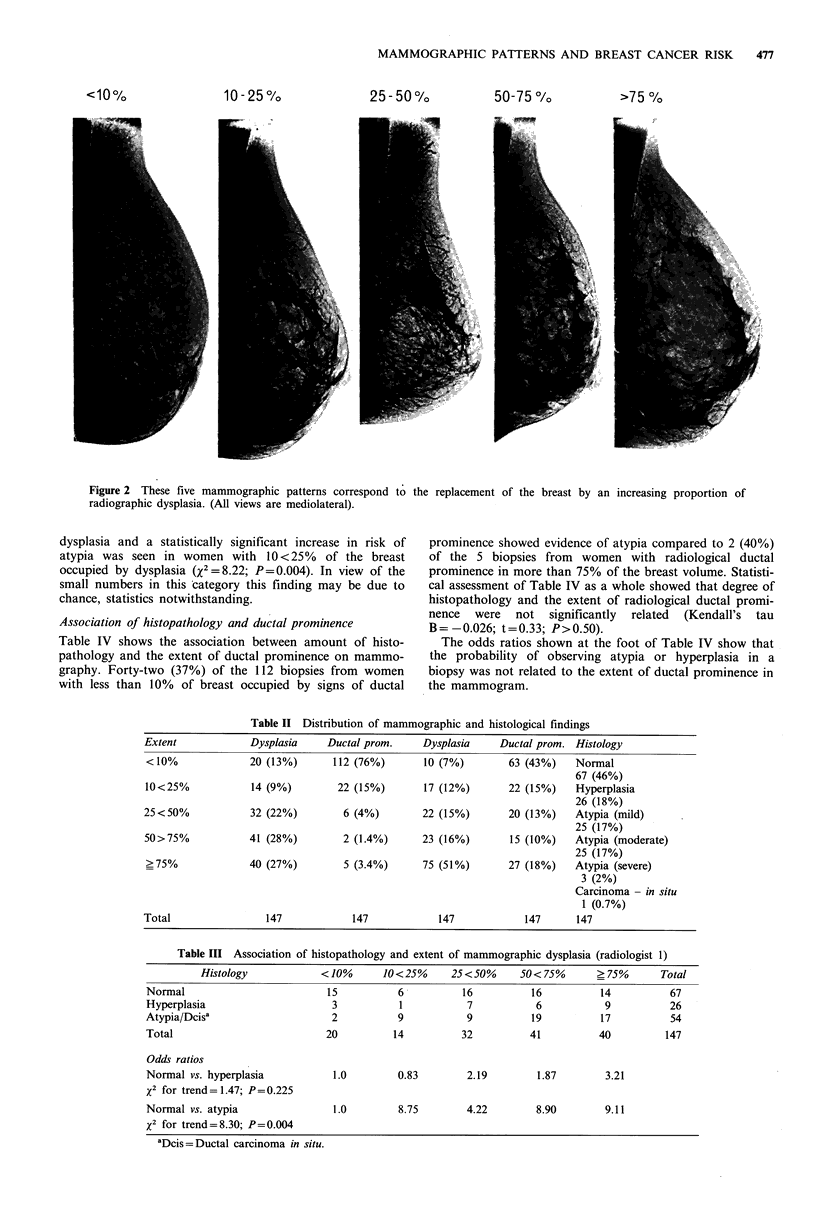

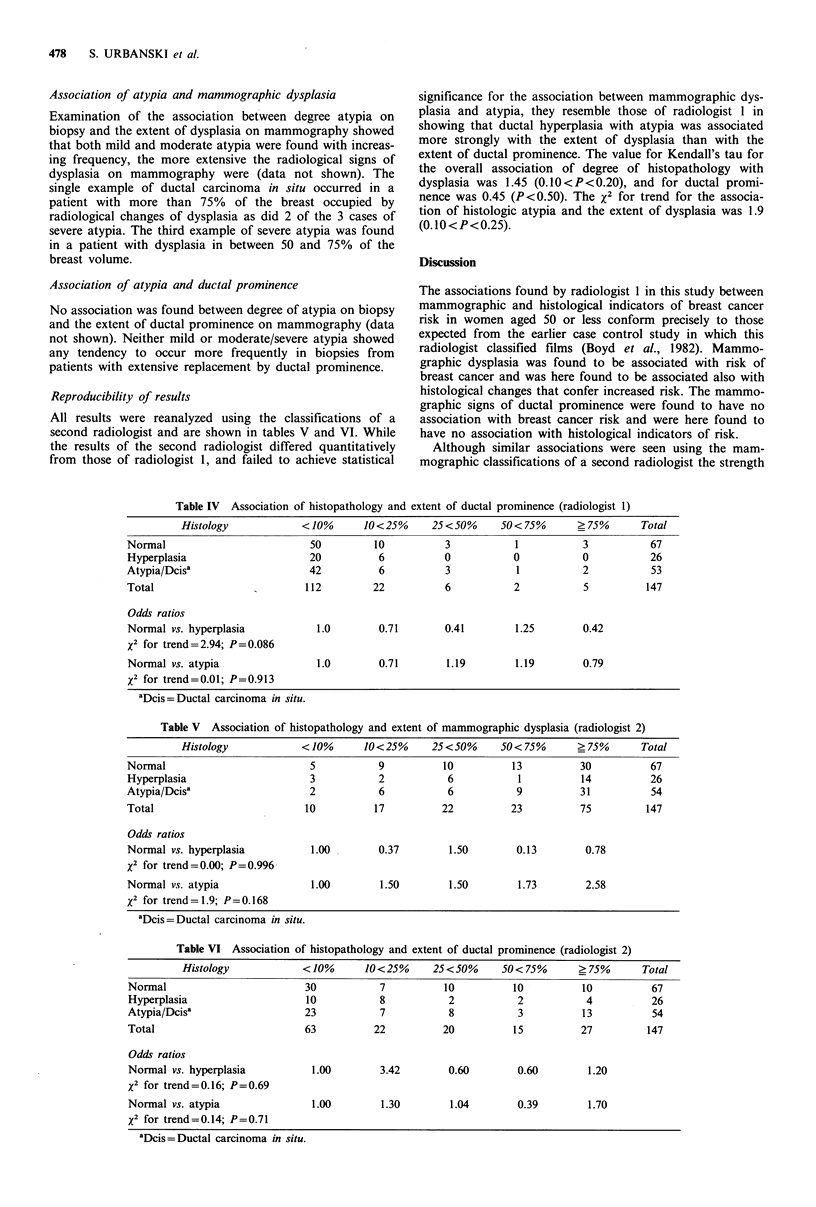

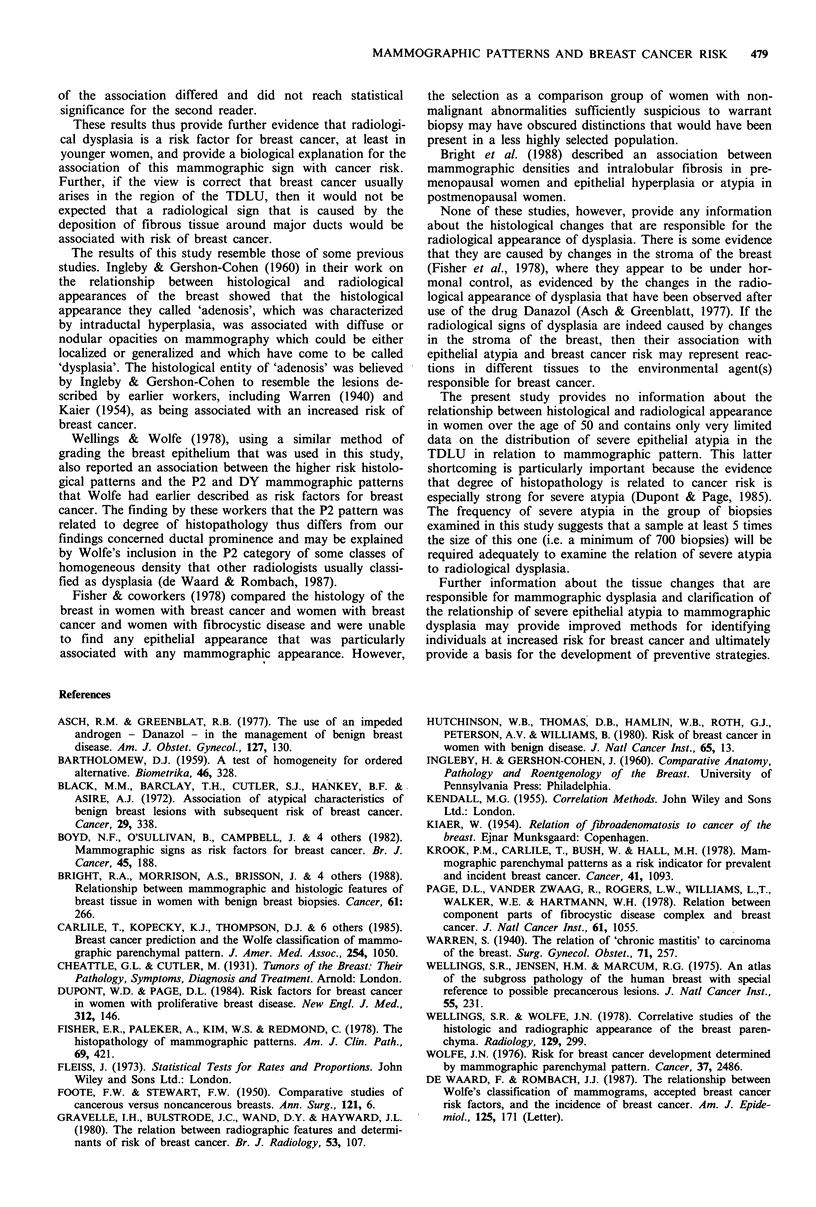


## References

[OCR_00591] Asch R. H., Greenblatt R. B. (1977). The use of an impeded androgen--danazol--in the management of benign breast disorders.. Am J Obstet Gynecol.

[OCR_00600] Black M. M., Barclay T. H., Cutler S. J., Hankey B. F., Asire A. J. (1972). Association of atypical characteristics of benign breast lesions with subsequent risk of breast cancer.. Cancer.

[OCR_00611] Bright R. A., Morrison A. S., Brisson J., Burstein N. A., Sadowsky N. S., Kopans D. B., Meyer J. E. (1988). Relationship between mammographic and histologic features of breast tissue in women with benign biopsies.. Cancer.

[OCR_00617] Carlile T., Kopecky K. J., Thompson D. J., Whitehead J. R., Gilbert F. I., Present A. J., Threatt B. A., Krook P., Hadaway E. (1985). Breast cancer prediction and the Wolfe classification of mammograms.. JAMA.

[OCR_00625] Dupont W. D., Page D. L. (1985). Risk factors for breast cancer in women with proliferative breast disease.. N Engl J Med.

[OCR_00630] Fisher E. R., Palekar A., Kim W. S., Redmond C. (1978). The histopathology of mammographic patterns.. Am J Clin Pathol.

[OCR_00639] Foote F. W., Stewart F. W. (1945). Comparative Studies of Cancerous Versus Noncancerous Breasts.. Ann Surg.

[OCR_00643] Gravelle I. H., Bulstrode J. C., Wang D. Y., Bulbrook R. D., Hayward J. L. (1980). The relation between radiographic features and determinants of risk of breast cancer.. Br J Radiol.

[OCR_00648] Hutchinson W. B., Thomas D. B., Hamlin W. B., Roth G. J., Peterson A. V., Williams B. (1980). Risk of breast cancer in women with benign breast disease.. J Natl Cancer Inst.

[OCR_00666] Krook P. M., Carlile T., Bush W., Hall M. H. (1978). Mammographic parenchymal patterns as a risk indicator for prevalent and incident cancer.. Cancer.

[OCR_00673] Page D. L., Vander Zwaag R., Rogers L. W., Williams L. T., Walker W. E., Hartmann W. H. (1978). Relation between component parts of fibrocystic disease complex and breast cancer.. J Natl Cancer Inst.

[OCR_00681] Wellings S. R., Jensen H. M., Marcum R. G. (1975). An atlas of subgross pathology of the human breast with special reference to possible precancerous lesions.. J Natl Cancer Inst.

[OCR_00687] Wellings S. R., Wolfe J. N. (1978). Correlative studies of the histological and radiographic appearance of the breast parenchyma.. Radiology.

[OCR_00692] Wolfe J. N. (1976). Risk for breast cancer development determined by mammographic parenchymal pattern.. Cancer.

[OCR_00696] de Waard F., Rombach J. J. (1987). Re: "The relationship between Wolfe's classification of mammograms, accepted breast cancer risk factors, and the incidence of breast cancer".. Am J Epidemiol.

